# Supplementary material for: Comparative Transcriptome Analysis Reveals Inhibitory Roles of Strigolactone in Axillary Bud Outgrowth in Ratoon Rice
Source: Plants (Basel). 2024 Mar 21;13(6):899. doi: 10.3390/plants13060899 (PMC10975295; doi:10.3390/plants13060899)
Supplement: Supplementary file 1 [file plants-13-00899-s001.zip › plants-2877912-Supplementary Figures3.1.pdf]

Supplementary Figures

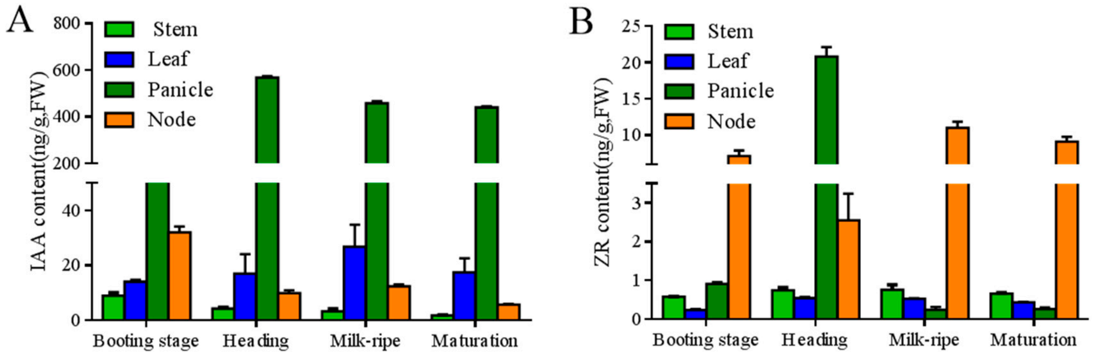

Figure S1. Changes of phytohormone contents in different organs of HHZ first seasonal rice. **A**, The IAA contents of first cropping HHZ rice in different periods. **B**, The contents of ZR of first cropping HHZ rice in different periods. Data represent means ± SE (*n*=3).

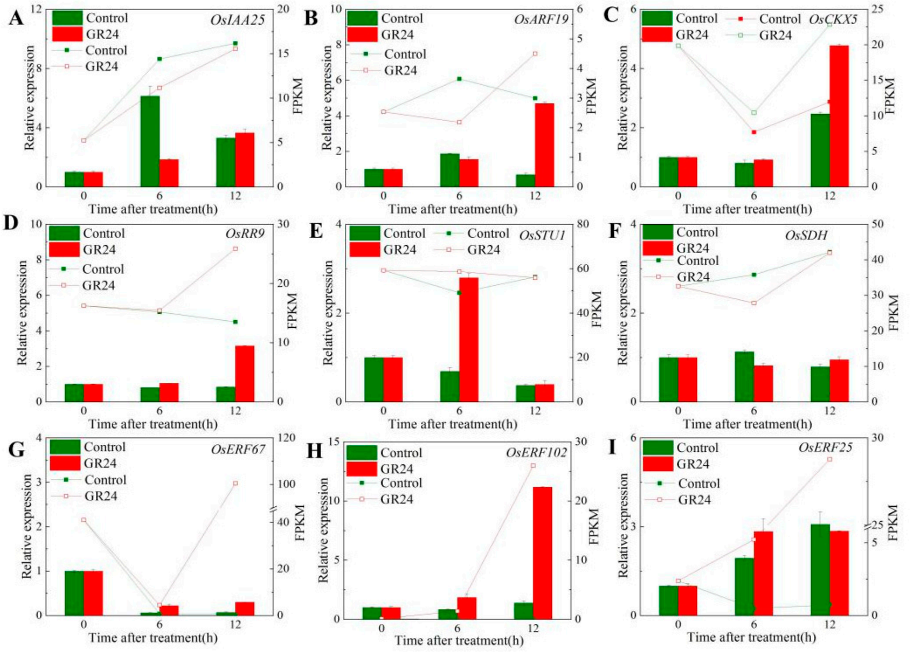

Figure S2. qRT-PCR validation for transcriptome data. Panels **A-I** represent the relative expression levels of *OsIAA25*, *OsARF19*, *OsCKX5*, *OsRR9*, *OsSUT1*, *OsSDH*, *OsERF67*, *OsERF102* and *OsERF25*, respectively. Data represent means ± SE (*n*=3). The histogram shows the relative expression level of qRT-PCR; Broken lines show the FPKM values of genes obtained by transcriptional sequencing. Expression value of 1 indicated that the gene expression level of 0 h as a control and convert the qPCR value of 0 h to 1. Symbols "*", "**" and "ns" indicated significant difference ($P<0.05$), extremely significant difference ($P<0.01$) and no significant difference, between GR24 treatment and control at the same treatment time, respectively.

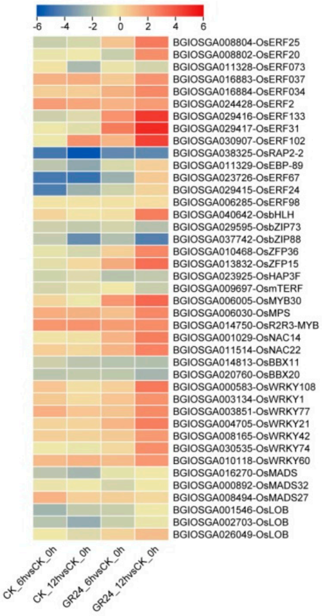

Figure S3. Analysis of differentially expressed genes annotated as transcription factors. A heatmap representing the relative expression of genes annotated as transcription factors. The relative expression levels are shown as log_2Foldchange levels. Blue cells indicate down-regulation and red cells indicate up-regulation.
